# Zinc Chloride: Time-Dependent Cytotoxicity, Proliferation and Promotion of Glycoprotein Synthesis and Antioxidant Gene Expression in Human Keratinocytes

**DOI:** 10.3390/biology10111072

**Published:** 2021-10-20

**Authors:** Beatriz Salesa, Roser Sabater i Serra, Ángel Serrano-Aroca

**Affiliations:** 1 Biomaterials and Bioengineering Lab, Centro de Investigación Traslacional San Alberto Magno, Universidad Católica de Valencia San Vicente Mártir, 46022 València, Spain; beatriz.salesa@ucv.es; 2 Centre for Biomaterials and Tissue Engineering, Universitat Politècnica de València, 46022 València, Spain; 3 Biomedical Research Networking Center, Bioengineering, Biomaterials and Nanomedicine (CIBER-BBN), 46022 Valencia, Spain

**Keywords:** biometals, zinc ions, human keratinocytes, cytotoxicity, gene expression, biomedical applications

## Abstract

**Simple Summary:**

Zinc ions are involved in the biology of cell growth, proliferation, differentiation or apoptosis by regulating many biological molecules, such as transcription factors, enzymes and growth factors. In this study, the time-dependent cytotoxicity, cell proliferation and gene expression in human keratinocytes HaCaT cells were evaluated when exposed to ZnCl_2_. The results of this study showed non-cytotoxic effects up to 10 µg/mL after 24 h, no significant effect on cell proliferation when exposed to 5 or 1 µg/mL ZnCl_2_ at 72 h and upregulation of eight genes, with great potential in the biomedical field, particularly for regenerative-medicine applications and wound healing.

**Abstract:**

The use of ionic metals such as zinc (Zn^2+^) is providing promising results in regenerative medicine. In this study, human keratinocytes (HaCaT cells) were treated with different concentrations of zinc chloride (ZnCl_2_), ranging from 1 to 800 µg/mL, for 3, 12 and 24 h. The results showed a time–concentration dependence with three non-cytotoxic concentrations (10, 5 and 1 µg/mL) and a median effective concentration value of 13.5 µg/mL at a cell exposure to ZnCl_2_ of 24 h. However, the zinc treatment with 5 or 1 µg/mL had no effect on cell proliferation in HaCaT cells in relation to the control sample at 72 h. The effects of the Zn^2+^ treatment on the expression of several genes related to glycoprotein synthesis, oxidative stress, proliferation and differentiation were assessed at the two lowest non-cytotoxic concentrations after 24 h of treatment. Out of 13 analyzed genes (superoxide dismutase 1 (*SOD1)*, catalase *(CAT)*, matrix metallopeptidase 1 *(MMP1),* transforming growth factor beta 1 *(TGFB1)*, glutathione peroxidase 1 (*GPX1),* fibronectin 1 (*FN1)*, hyaluronan synthase 2 *(HAS2)*, laminin subunit beta 1 *(LAMB1),* lumican *(LUM)*, cadherin 1 (*CDH1)*, collagen type IV alpha *(COL4A1)*, fibrillin (*FBN)* and versican *(VCAN*)), Zn^2+^ was able to upregulate *SOD1, CAT, TGFB1, GPX1, LUM, CDH1, FBN* and *VCAN*, with relative expression levels of at least 1.9-fold with respect to controls. We found that ZnCl_2_ promoted glycoprotein synthesis and antioxidant gene expression, thus confirming its great potential in biomedicine.

## 1. Introduction

Zinc ions (Zn^2+^) are involved in all the crucial decisions in the life of mammalian cells related to growth, proliferation, differentiation or apoptosis, both in its ionic or protein-bound form [1]. They are implicated in regulating many biological molecules, such as transcription factors, enzymes and growth factors [2,3], and have been shown to be crucial in hundreds of enzymatic reactions and required for thousands of transcription factors which regulate gene expression [4]. Zn^2+^ has also been recently recognized as an intra- and intercellular signaling mediator, acting similar to calcium as a second messenger to transduce extracellular stimuli into intracellular signaling events [3,4].

Zn^2+^, with a mass of 0.8–3 g in the human body, can be found in tissues such as muscle and bone, which act as a major tissue reservoir (85% of the whole body); skin (5%); liver (5%); and the remaining percentage is distributed around other organs, such as the brain, pancreas and kidneys. Zn^2+^ deficiency is related to delayed bone development and dwarfism; in addition, several skin disorders are associated with Zn^2+^ deficiency [4,5,6,7].

The essential role played by Zn^2+^ ions in cell behavior, modulating cell signaling, has attracted increasing attention in the biomedical field, particularly for regenerative-medicine applications and wound healing [8,9,10,11,12,13,14,15]. In addition, Zn^+2^ presents antimicrobial activity, both as an antibacterial and antifungal agent [16,17]. However, it has been reported that, to achieve effective antimicrobial activity on common wound pathogens, such as *Staphylococcus aureus, Escherichia coli, Pseudomonas aeruginosa* and *Candida albicans*, higher concentrations of Zn compounds are required than those used to promote cell response [18]. In that study, doses of zinc sulfate and zinc gluconate lower than 10 µg/mL showed no antimicrobial properties, but they exerted bioactivity in nutrient-deficient environments.

Regenerative medicine involves the regeneration of human tissues, with the aim of returning the patient to full health, using bioactive factors to act in conjunction with the natural healing potential [19]. Among the bioactive factors that have shown a great potential, metallic ions (including Zn^2+^) have emerged, based on their unique properties to modulate cell response [12]. Zn^2+^-based biomaterials have recently been developed, including bioactive glasses [8,9], nanocomposites with ZnO nanoparticles [20] and hydrogels [21,22,23,24]. Mesoporous bioactive glasses containing Zn^2+^ have been reported as potential biomaterials for soft-tissue repair and wound healing [9], whilst ZnO nanoparticles with different nanostructures have been shown to promote adhesion, growth and differentiation in several cell lines [25,26]. Nanocomposite hydrogels based on alginate/graphene oxide [21] or carboxymethylcellulose/ZnO [24] crosslinked with Zn^2+^ ions have been prepared to provide bioactivity and antimicrobial properties. Zn^2+^ ions have been shown to promote tissue formation and inhibit resorption in musculoskeletal disorders [27].

Zn^2+^-based compounds such as calamine or zinc oxide have been applied topically for centuries to sooth and calm skin irritations and enhance wound healing [28]. Its use has also expanded over the years for several dermatological conditions, including infections inflammatory dermatoses, pigmentary disorders and neoplasia. Therapeutically, Zn^2+^ can be used both topically and in systemic form; however, systemic Zn^2+^ therapy needs further experimental and clinical evidences [28,29]. Zn^2+^ plays an important physiological function throughout the stages of wound healing, associated with inflammation and immune response [4,30,31]. The importance of Zn^2+^ concentration for wound healing has been reported in patients with thermal injuries or exposure to surgical stress [15]. In human skin, the epidermis contains higher concentrations of Zn^2+^ than the dermis (60 µg/g vs. 40 µg/g), due to a Zn^2+^ requirement for epidermal keratinocytes proliferation and differentiation [32]. Keratinocytes are the main cell components of the epidermis (95%), with several key function in the wound-healing process. The signaling interaction between keratinocytes and other cells that participate in wound healing is crucial to wound closure [33]. Zn^2+^ therapies have been used in wound care to promote healing in patients with Zn^2+^ deficiency, as has topical zinc sulfate or zinc chloride (ZnCl_2_), due to its antioxidant effect [15]. Zn^2+^ is crucial for normal skin function and wound healing, acting as a bioactive factor mimicking the action of growth factors by promoting intracellular mitogenic signaling pathways [34]. It also acts as a stabilizer of the cell membrane of keratinocytes, being found both intracellularly and within the skin extracellular matrix [35]. However, high concentrations of extracellular Zn^2+^ ions have cytotoxic effects, due to the balance between extracellular–intracellular Zn^2+^ concentration and cell survival [35]. Thus, whilst Zn^2+^-based therapy is a promising tool in wound healing and dermatological diseases, a deeper understanding of the cellular behavior induced by Zn^2+^ is still needed in order to identify the boundaries that limit its safe therapeutic application. Therefore, the aim of this study was to analyze the biological response in terms of time-dependent cytotoxicity, cell proliferation and gene expression in human keratinocytes HaCaT cells when exposed to extracellular Zn^2+^(from ZnCl_2_). Gene expression associated with glycoprotein synthesis, oxidative stress, proliferation and differentiation was investigated for the first time to obtain further insight into the physiological roles and mechanisms of Zn^2+^ action.

## 2. Materials and Methods

### 2.1. Materials

ZnCl_2_ (≥97.0%, bioreagent for molecular biology suitable for cell culture) and 3-(4,5-dimethylthiazol-2-yl)-2,5-diphenyltetrazolium bromide (MTT) were acquired from Sigma-Aldrich (St. Louis, MO, USA). Dulbecco’s modified Eagle’s medium (DMEM), fetal bovine serum (FBS), L-Glutamine, penicillin–streptomycin (P/S), phosphate-buffered saline (PBS), trypsin–EDTA, dimethyl sulfoxide (DMSO) and epidermal growth factor were purchased from Life Technologies (Gibco, Karlsruhe, Germany).

### 2.2. Preparation of ZnCl_2_ Stock Solution

A stock solution of ZnCl_2_ was prepared in sterile DMEM low glucose supplemented with P/S and L-Glutamine by sonication for 2 h to dissolve the salt completely and obtain a homogeneous solution. The solution was used immediately after the sonication process as source of Zn^2+^ for in vitro cytotoxicity experiments.

### 2.3. Cell Culture

Immortalized human keratinocytes (cell line HaCaT) were supplied by the Medical Research Institute Hospital La Fe (Valencia, Spain). They were cultured in DMEM low glucose, containing l-glutamine, supplemented with 10% FBS and 1% P/S (complete medium) in a humidified atmosphere, at 5% CO_2_ and 37 °C. Culture medium was changed every 2 days, and cells were trypsinized for 3 min and resuspended in the same medium when culture achieved 80% confluence.

### 2.4. Cell Viability Assay

Cytotoxicity was assessed for keratinocytes exposed to different Zn^2+^ concentrations by performing an MTT assay [36], based on the metabolic reduction of the tetrazolium dye to a colored compound (formazan) through the mitochondrial succinate dehydrogenase. Cells were trypsinized and resuspended in complete medium, centrifuged and resuspended again in medium with 1% P/S and L-Glutamine, but without FBS, in order to avoid any chemical interaction with the zinc divalent cations. Then 96-micro-well plates were seeded at a low density (10^4^ cells/well) and placed in the incubator. After 24 h to allow cell adhesion, the culture medium was substituted by growth medium supplemented with 100 µL of ZnCl_2_ solution at concentrations between 1 and 800 µg/mL of Zn^2+^. Cells with medium without Zn^2+^ were used as control, and culture medium was used as reagent blank. Cytotoxicity was measured at different end points: 3, 12 and 24 h. After the exposure time, the culture medium was removed, and 100 µL of MTT working solution was added to each well. Plates were incubated for 3 h, at 37 °C. After removing the reagent solution and rinsing with PBS, we added 100 µL of DMSO to dissolve the formazan crystals and incubated for 1 h, at 37 °C. The absorbance of the resulting solution was measured at 550 nm to determine cell viability, using the Varioskan Lux microplate reader (ThermoScientific^TM^, Dreieich, Germany). Median effective concentration (EC_50_ assay) was obtained for ZnCl_2_ concentrations 1, 14, 16 and 18 µg/mL (see Results). EC_50_ values were calculated as the concentration where the sigmoidal curve attains values of 50%. Experiments were performed in sextuplicate on the same plate.

### 2.5. Proliferation Assay

Cell proliferation was determined by using the MTT assay [36] 72 h after ZnCl_2_ treatment. Following the same protocol described to assess cell viability, 5·10^3^ cells/well were seeded in a 96-multi-well culture plate. After 24 h, the growth medium was replaced by DMEM+1%P/S medium supplemented with 0.5%FBS to avoid complete starvation [37], together with ZnCl_2_ at specific concentrations, and cultured in a humidified atmosphere of 5% CO_2_ and 37 °C. As positive control, cells treated with growth medium (DMEM+1%P/S+0.5%FBS) and were supplemented with Epidermal Growth Factor (EGF) at a concentration of 15 ng/mL. Moreover, a control group with only growth medium was included. Based on the cell viability assay results, two non-cytotoxic concentrations of ZnCl_2_ at 24 h were chosen (1 and 5 µg/mL) to perform this assay. Experiments were performed in sextuplicate on the same plate.

### 2.6. Gene Expression

Real-time reverse-transcription polymerase chain reaction (RT-qPCR) was carried out to analyze the gene expression in HaCaT cells upon treatment with extracellular Zn^2+^. Cells were seeded at a density of 1.5·10^5^ cells/well in a 6-well plate and cultured in a CO_2_ incubator, following the same protocol used in the cytotoxicity assays. After 24 h, cells were treated with two non-cytotoxic concentrations of ZnCl_2_ (1 and 5 µg/mL), i.e., the same used in the proliferation assay, and cultured for 24 h. After removing culture medium and rinsing with PBS, we added the extraction solution, and the plates were frozen in liquid nitrogen. RNA purification kit (Norgen, Thorold, ON, Canada) was used for RNA isolation, following the protocol provided by the manufacturer. The quality and the concentrations of the different samples were determined by using a Nanodrop™ One (ThermoScientific™, Dreieich, Germany) and a PrimeScript™ RT Reagent Kit (Perfect Real Time) (Takara Bio, Inc., Kusatsu, Japan) to synthetized cDNA. Quantitative PCR, TB Green Premix Ex Taq (Takara Bio, Inc.), was performed by following the protocol provided by the manufacturer in a 384 QuantStudio 5 (ThermoScientific™, Dreieich, Germany). Data analysis was carried out by using QuantStudio^TM^ software. Primers of target genes and the reference gene (β-actin/ACTB), which are reported in Table 1, were measured by using Primer-Blast software (available on: http://www.ncbi.nlm.nih.gov/tools/primer-blast, accessed on 18 October 2021). Data normalization was performed based on the reference gene expression. Experiments were performed in triplicate on the same plate.

### 2.7. Statistical Analysis

Data were reported as mean ± standard deviation (SD). Statistical analysis was conducted through GraphPad Prism 6 software (GraphPad Inc., San Diego, CA, USA). Statistical differences were tested by one-way ANOVA with Tukey’s correction for multiple comparisons. The EC_50_ values were determined by using Probit analysis. Significance was assumed at *p*-values < 0.05 (95% confidence).

## 3. Results and Discussion

### 3.1. Cytotoxicity Assay

**Figure 1:**
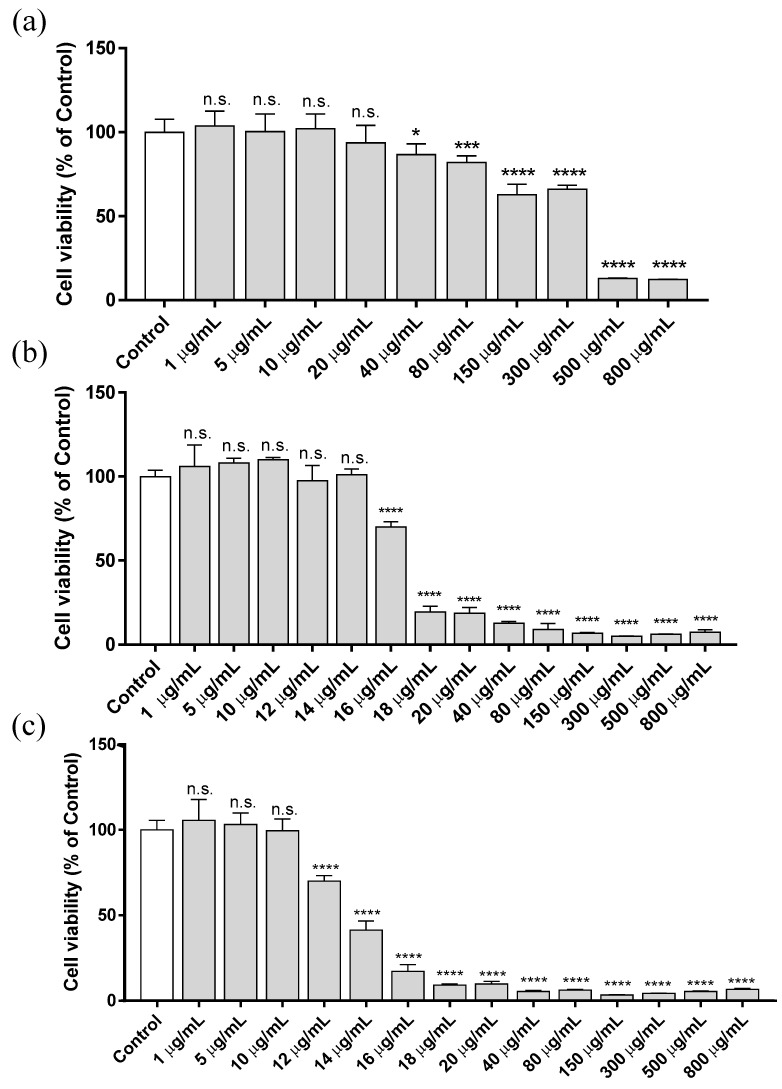
Cytotoxicity assay (MTT method) in human keratinocytes HaCaT cell line after exposing the cells for 3 h (**a**), 12 h (**b**) and 24 h (**c**) to several concentrations of ZnCl_2_, ranging from 1 to 800 µg/mL. Results represented are normalized to the control group (culture media without ZnCl_2_). Data depicted as mean ± SD of six replicates. Significant differences with respect to control were determined by one-way ANOVA with Tukey’s correction for multiple comparisons: * *p* > 0.05; *** *p* > 0.001; **** *p* > 0.0001; n.s. = not significant.

To evaluate the cytotoxicity of ZnCl_2_, HaCaT cells were cultured in culture medium supplemented with increasing concentrations of ZnCl_2_, ranging from 1 to 800 µg/mL. The results were assessed at different exposure times (3, 12 and 24 h), and cell viability was determined by using the MTT-based colorimetric assay. Figure 1 shows the cytotoxicity results at 3, 12 and 24 h after exposure.

The EC_50_ calculated for each exposure time is reported in Table 2. The highest cytotoxic effect due to ZnCl_2_ occurs within the first 12 h of exposure. The EC_50_ at 12 h was more than 90% lower than that of the concentration obtained after 3 h of treatment. However, between 12 and 24 h of treatment, there was only a 20% reduction in EC_50_. In fact, it was only possible to detect this difference in EC_50_ between 12 and 24 h with the addition of several concentrations between 10 and 20 µg/mL. These results indicate that the main cytotoxic effect of Zn^2+^ added to the culture medium takes place in the first hours of exposure.

Zn^2+^-induced cytotoxicity depends on the cell line [38]. Studies in human lens epithelial cell line (HLE B-3) have shown a high ZnCl_2_ sensitive dose-dependent response, where the cytotoxic effect was obtained with 5 µg/mL of ZnCl_2_ after 72 h of treatment [39]. Evident cytotoxicity produced by ZnCl_2_ exposure was observed in C6 rat glioma at a concentration of 25 µg/mL (24 h after the treatment) [40]. In vivo assays performed in Wistar rats showed that concentrations below 10 mg/kg did not induce mortality in the exposed animals. An increase in lethality was observed when the dose concentration was increased, inducing a 10% mortality at 20 mg/kg body weight, with 60 mg/kg of LC_50_ (lethal concentration 50) [41]. In that study, a drastic reduction in cell viability was observed with an in vitro concentration of 20 µg/mL at 12 and 24 h of exposure, which is in the same order of magnitude as our results. Other Zn^2+^-based compounds, such as ZnO, induced higher toxicity in laryngeal cells (viability lower than 40%) at a concentration of 10 µg/mL after 24 h of exposure [42].

### 3.2. Proliferation Assay

**Figure 2:**
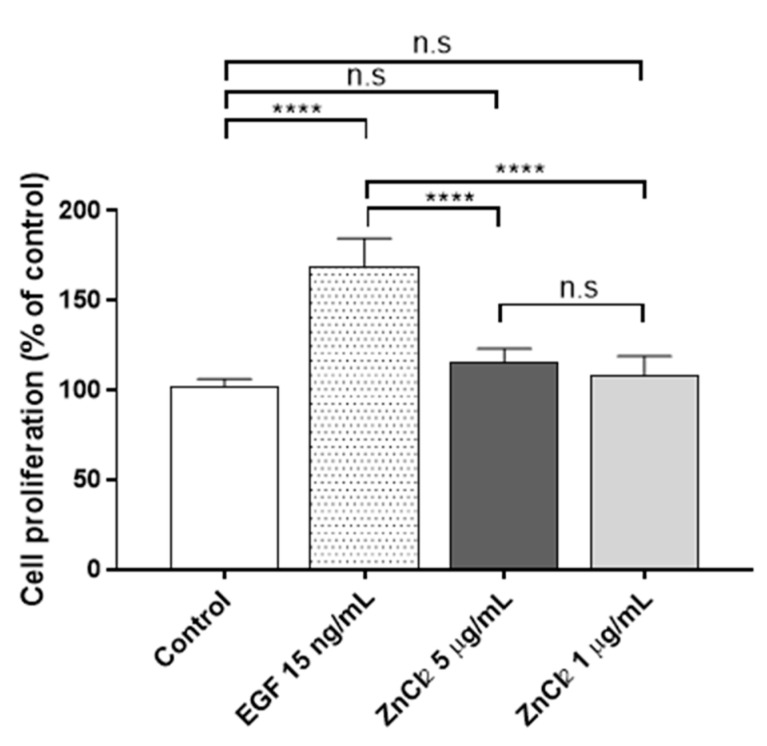
Proliferative activity of ZnCl_2_ after 72 h of exposure in human keratinocytes. Results represented are normalized to the control group (culture media without ZnCl_2_). Data depicted as mean ± SD of six replicates. Significant differences were determined by one-way ANOVA with Tukey’s correction for multiple comparisons: **** *p* > 0.0001; n.s. = not significant.

In the cytotoxicity assay, it was found that Zn^2+^ concentration became cytotoxic at concentrations from 12 µg/mL onwards after 24 h of Zn^2+^ exposure. Thus, two non-cytotoxic ZnCl_2_ concentrations were chosen for the proliferation assays, namely 1 and 5 µg/mL, to minimize the cytotoxic effect at a longer time (72 h). Thus, proliferation was assessed after 72 h of ZnCl_2_ exposure in human keratinocytes (Figure 2).

In both concentrations, the zinc treatment had no significant effect on cell proliferation after 72 h of culture. In a previous study [43], it was found that a higher concentration of extracellular ZnCl_2_ (100 µM (~13 µg/mL)) induced slight but significant cell proliferation after 72 h of exposure (1.3-fold; *p* = 0.045) in HaCaT cells. In the present study, concentrations of extracellular Zn^2+^ higher than 12 µg/mL were found to show a slight cytotoxicity after 24 h of treatment and therefore were discarded for the proliferation assays. In good agreement with our results, Emri et al. [43] showed that a close extracellular concentration of Zn^2+^ 50 µM (~6.5 µg/mL) had no effect on cell proliferation in HaCaT cells. However, many controversial results have been published in the literature with regards to the toxicity of Zn^2+^ ions [35], and further investigation is therefore required. It should be noted that the culture media, as well as the different techniques used in the experiments, can give rise to differences in the outcomes. In Holmes’s study [35], the researchers found that the results from the cytotoxicity assay, performed after 24 h of the Zn^2+^ treatment in HaCaT cells with extracellular ZnSO_2_, depended on the medium used in the cell culture: complete DMEM, complete DMEM with 1.75% bovine serum albumin (BSA) or complete DMEM with 1 mM Ethylenediaminetetraacetic acid solution (EDTA). Both BSA and EDTA can act as Zn^2+^ chelators, reducing the Zn^2+^ toxicity. The techniques used in the cytotoxic studies (EZ4U or MTS assays) also affect the results [35,43].

### 3.3. Gene Expression

The protective effect of non-cytotoxic ZnCl_2_ concentrations against UVB radiation was studied in human epidermal keratinocytes [43]. Although the data showed a reduction on pyrimidine dimers in cells pretreated with ZnCl_2_, significantly enhanced superoxide generation was found 10 h after UVB treatment. The results of that study therefore suggested that the exposure of human keratinocytes to non-toxic concentrations of zinc chloride impacts gene expression, cell proliferation and the responses to external stress (such as UVB radiation) in the skin. It was also demonstrated that primary keratinocytes exposed to nM concentration of zinc pyrithione (ZnPT) loosed genomic integrity [44]. Exposure of HaCaT cells to ZnO nanoparticles induces the expression of genes involved in oxidative stress [45]. SOD genes were significantly higher in cells exposed to Zn nanoparticles, suggesting its potential to induce intracellular reactive oxygen species (ROS) and oxidative stress.

**Figure 3:**
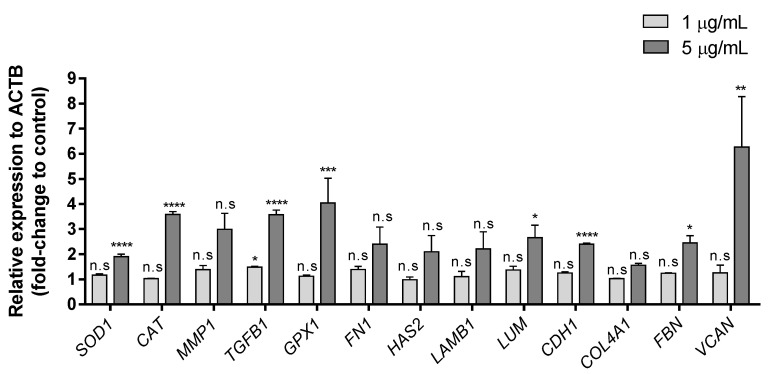
Effects of Zn^2+^ on HaCaT cell line after 24-h treatment. Expression of different genes at 1 and 5 µg/mL ZnCl_2_ concentration. Data depicted as mean ± SD of three replicates. Results represented as fold-change to control and relative expression to ACTB. Significant differences were determined by one-way ANOVA with Tukey’s correction for multiple comparisons: * *p* < 0.05; ** *p* < 0.01; *** *p* < 0.001; **** *p* > 0.0001; n.s. = not significant.

To determine the effects of exposure to extracellular Zn^2+^ ions on the expression of genes involved in different metabolic routes, thirteen genes, which are related to the glycoprotein synthesis associated with repair and maintenance of different tissues, oxidative stress and damage, proliferation, differentiation and cell growth, were analyzed (Table 1). Figure 3 shows the results obtained after analyzing gene expression in HaCaT cells treated for 24 h with 1 or 5 µg/mL of extracellular Zn^2+^, referred to the housekeeping gene (β-actin/ACTB).

The lowest concentration (1 µg/mL) did not show significant differences in any of the genes analyzed (only *TGFB1* showed a slight upregulation). Nevertheless, exposure to 5 µg/mL induced significant upregulation in eight genes (*SOD1, CAT, TGFB1, GPX1, LUM, CDH1, FBN* and *VCAN*) with relative expression levels of at least 1.9-fold with respect to untreated controls. *VCAN* gene showed the highest overexpression after the extracellular Zn^2+^ treatment (6.265-fold). This gene is involved in cell adhesion, proliferation, migration and angiogenesis and plays an important role in tissue morphogenesis and maintenance [46]. It has also been reported that keratinocytes express versican during active cell proliferation [47]. The expression on this gene was upregulated in association with healing/restructuring processes following non-surgical periodontal treatments [48]. In addition, cell exposure to ZnCl_2_ at a concentration of 5 µg/mL upregulated the genes involved in glycoprotein synthesis. Gene expression of *FBN*, *LUM* and *CDH1* was upregulated (2-fold). Type I cadherins includes vital transmembrane glycoproteins for the morphogenesis and development of normal animal tissue [49]. Studies carried out on UV-irradiated HaCaT cells showed how the decrease in the synthesis of these glycoproteins compromises cell–cell adhesion in epithelial tissues, thus compromising the integrity of the skin after radiation [50]. Moreover, *LUM* is the major keratan sulfate proteoglycan of the cornea, and it is distributed throughout the body in interstitial collagenous matrices. It has been reported in this cell line that lumican is involved in inflammatory cells infiltration [51] and angiogenesis [52], which are essential in wound healing. Studies performed on fibroblasts and keratinocytes demonstrated that lumican can modulate fibroblast activation via integrin a2, whereas keratinocytes were not affected [53]. In our study, *LUM* gene expression was activated by the exposure to zinc chloride, which could lead to triggering the signaling cascade and subsequent activation of cells in the lower layers of the skin. *FBN* is an extracellular matrix glycoprotein that provides force-bearing structural support in elastic and non-elastic connective tissue throughout the body and serves as a structural component of calcium-binding microfibrils [54]. It has also been related to growth-factor signaling, being crucial for correct growth and balance of skin homeostasis [55]. During metabolic processes, ROS are present due to the requirements for normal cell signaling. In the same way as with lumican, the expression of fibrillin increased in fibroblasts after treatment with xanthohumol, which improves the structure and firmness of the skin [56]; moreover, it has also been demonstrated that these glycoproteins can be synthetized by HaCaT cells [57]. However, high ROS levels cause severe cellular damage and may lead to the development of skin diseases, including cancer [58,59]. Induction of gene expression involved in oxidative stress could protect cells against oxidative cellular damage in the presence of different external stressors. Vitamin E has been used as antioxidant to pre-protect human epidermal keratinocytes and enhance their therapeutic ability under induced oxidative microenvironments [60]. Other exogenous antioxidants have also been reported recently, such as extract of *Rhodiola rosea* L. roots [61], cannabidiol [62] and other different antioxidant agents [63]. Recently, a compound of nicotinamide, a soluble vitamin B_3_ well-known as an antioxidant, and a jellyfish peptide (nicotinyl–isoleucine–valine–histidine peptide) have also been reported to promote antioxidant gene expression in HaCaT Cells [64].

*CAT*, *GPX1* and *TGFB1* were upregulated with fold-changes between 3 and 4. *CAT* and *GPX1* genes act as relevant antioxidants, encoding the synthesis of enzymes related in the neutralization of H_2_O_2_. UVA and UVB irradiation causes biological damage by inducing an increase in ROS levels in human keratinocytes [65,66], causing cellular oxidative stress, damage to cell components and genomic instability [67,68]. UVB induces an increase in ROS levels in two stages, immediately following UVB irradiation and several hours after irradiation [69]. It has been shown that *CAT* overexpression has a protective role against UVB irradiation by preventing DNA damage mediated by the late ROS increase [66]. In addition, UVA induces upregulation of the transcription factor AP-2α, involved in epidermal differentiation, which can be attenuated by the overexpression of *GPX1* [65]. Both *CAT* and *GPX1* were upregulated after extracellular Zn^2+^ treatment for 24 h at a concentration of 5 µg/mL. Exposure to 1 µg/mL did not show any change in gene regulation; thus, a higher concentration exposure is required to activate these defense mechanisms in HaCaT cells. In addition, previous studies in the same cell line treated with caffeic acid or ferulic acid [70] concluded that this upregulation could result in a protective effect on cells against oxidative stressors. *TGFB 1*, a well-established melanoma suppressor gene [71], which is also involved in cell proliferation, differentiation and growth regulation, was also activated when cells were treated with the highest extracellular Zn^2+^ concentration (5 µg/mL).

The protein encoded by *SOD1* gene binds copper and zinc ions and it is one of two isozymes responsible for destroying free superoxide radicals in the body. *SOD 1* is the representative antioxidant enzyme among all SOD enzymes. It can be activated by different substances, such as genoposidic acid [72], sinapic acid [73], or nicotinyl–isoleucine–valine–histidine [64]. However, other promising compounds such as graphene oxide, are not able to upregulate the *SOD1* gene using non-cytotoxic concentrations [74]. In the present study, *SOD1* was upregulated with extracellular Zn^2+^ at the highest concentration (5 µg/mL), suggesting that non-cytotoxically extracellular Zn^2+^ concentrations are able to induce an antioxidant effect in HaCaT keratocytes.

Therefore, we have shown that ZnCl_2_ could be a promising bioactive antioxidant against external factors and promote skin regeneration in wound healing or skin-disease therapeutics, thus confirming its great potential in biomedicine. However, further research is required to fully assess the efficacy and safety before clinical use.

## 4. Conclusions

The results show a time-dependent cytotoxity of ZnCl_2_ with non-cytotoxic concentrations up to 10 µg/mL in human keratinocyte HaCaT cells treated for 3, 12 and 24 h. However, 5 µg/mL of ZnCl_2_ induced significant upregulation in eight genes (*SOD1, CAT, TGFB1, GPX1, LUM, CDH1, FBN* and *VCAN*), with relative expression levels of at least 1.9-fold with respect to untreated controls. ZnCl_2_ promoted glycoprotein synthesis and antioxidant gene expression in this cell line. Therefore, we have shown that ZnCl_2_ could be a promising antioxidant compound against external factors. It could be used as a bioactive agent and promoter of regeneration in wound healing or skin-disease treatments. This study demonstrates the great potential of Zn^2+^ ions in the form of ZnCl_2_ salt in biomedical applications.

## Figures and Tables

**Table 1:** Details of specific genes used in the RT-qPCR assay.

Gene Symbol (Access Number)	Gene Name	Oligo Sequences	Function
**ACTB (NM_001101)**	Actin beta	5′-CCATGCCCACCATCACGC-3′	Highly conserved protein that are involved in cell motility, structure and integrity.
		5′-CACAGAGCCTCGCCTTTG-3′	
**CAT (NM_001752)**	Catalase	5′-TGAATGAGGAACAGAGGAAACG-3′	Encodes a key antioxidant enzyme (catalase) in the body defense against oxidative stress.
		5′-AGATCCGGACTGCACAAAG-3′	
**MMP1 (NM_001145938)**	Matrix metallopeptidase 1	5′-GGACCATGCCATTGAGAAAG-3′	Involved in the breakdown of extracellular matrix in normal physiological processes.
		5′-TCCTCCAGGTCCATCAAAAG-3′	
**GPX1 (NM_000581)**	Glutathione peroxidase 1	5′-TTTGGGCATCAGGAGAACGC-3′	Catalyze the reduction of organic hydroperoxides and hydrogen peroxide by glutathione, and thereby protect cells against oxidative damage.
		5′-ACCGTTCACCTCGCACTTC-3′	
**COL4A1 (NM_000088)**	Collagen type I alpha 1	5′-CAAGGGCGACAGAGGTTTGC-3′	Abundant in bone, cornea, dermis and tendon. Mutations in this gene are associated with osteogenesis imperfect types I–IV.
		5′-AAAACTCACCAGGCTCCCCC-3′	
**TGFB1 (NM_000660)**	Transforming growth factor beta 1	5′-AGCTGTACATTGACTTCCGCA-3′	Regulates cell proliferation, differentiation and growth.
		5′-TGTCCAGGCTCCAAATGTAGG-3′	
**HAS2 (NM_005328)**	Hyaluronan synthase 2	5′-CCGAGAATGGCTGTACAATGC-3′	Involved in a variety of functions, such as space filling, lubrication of joints and provision of a matrix through which cells can migrate.
		5′-AGAGCTGGATTACTGTGGCAA-3′	
**LAMB1 (NM_002291)**	Laminin subunit beta 1	5′-CAGGGTGTGCAGTCAGGGAA-3′	Implicated in a wide variety of biological processes, including cell adhesion, differentiation, migration, signaling, neurite outgrowth and metastasis.
		5′-TGTGTCTGCGTTGAGGGTGT-3′	
**LUM (NM_002345)**	Lumican	5′-ACTTGGGTAGCTTTCAGGGCA-3′	Is the major keratan sulfate proteoglycan of the cornea, but is also distributed in interstitial collagenous matrices throughout the body.
		5′-TTCCTGGCATTGATTGGTGGT-3′	
**FN1 (NM_001306129)**	Fibronectin 1	5′-GGCCAGTCCTACAACCAGT-3′	Involved in cell adhesion and migration processes, including embryogenesis, wound healing, blood coagulation, host defense and metastasis.
		5′-CGGGAATCTTCTCTGTCAGC-3′	
**VCAN (NM_001126336)**	Versican	5′-CTGGTCTCCGCTGTATCCTG-3′	Involved in cell adhesion, proliferation, migration and angiogenesis and plays a central role in tissue morphogenesis and maintenance.
		5′-ATCGCTGCAAAATGAACCCG-3′	
**CDH1 (NM_001317184)**	Cadherin 1	5′-AACAGCACGTACACAGCCCT-3′	Loss of function of this gene is thought to contribute to cancer progression by increasing proliferation, invasion and/or metastasis.
		5′-TCTGGTATGGGGGCGTTGTC-3′	
**FBN (NM_000138)**	Fibrillin 1	5′-ATCCAACCACGTGCATCAGT-3′	Extracellular matrix glycoprotein that serves as a structural component of calcium-binding microfibrils, providing force-bearing structural support in elastic and non-elastic connective tissue throughout the body.
		5′-AGAGCGGGTATCAACACAGC-3′	
**SOD1 (NM_000454)**	Superoxide dismutase 1	5′-GGTGTGGCCGATGTGTCT-3′	The protein encoded by this gene binds copper and Zn^2+^ ions and is one of two isozymes responsible for destroying free superoxide radicals in the body.
		5′-TCCACCTTTGCCCAAGTCA-3′	

**Table 2:** Exposure of human keratinocyte HaCaT cells to ZnCl_2_ for 3, 12 and 24 h. Median effective concentration (EC_50_), confidence limits 95% (CI) as mass/volume in µg/mL and R square.

ZnCl_2_ Exposure	EC_50_ (µg/mL)	95% CI	R Square
3 h	193.1	172.6–216.5	0.9524
12 h	16.8	16.5–17.1	0.9598
24 h	13.5	13.2–13.6	0.9799

## Data Availability

Data are contained within the article.

## References

[1] Beyersmann D., Haase H. (2001). Functions of zinc in signaling, proliferation and differentiation of mammalian cells. BioMetals.

[2] Fukada T., Kambe T. (2019). Zinc Signaling.

[3] Hara T., Takeda T.A., Takagishi T., Fukue K., Kambe T., Fukada T. (2017). Physiological roles of zinc transporters: Molecular and genetic importance in zinc homeostasis. J. Physiol. Sci..

[4] Ogawa Y., Kawamura T., Shimada S. (2016). Zinc and skin biology. Arch. Biochem. Biophys..

[5] Jackson M.J. (1989). Physiology of Zinc: General Aspects.

[6] Tapiero H., Tew K.D. (2003). Trace elements in human physiology and pathology: Zinc and metallothioneins. Biomed. Pharmacother..

[7] Ogawa Y., Kinoshita M., Shimada S., Kawamura T. (2018). Zinc and skin disorders. Nutrients.

[8] Zamani D., Moztarzadeh F., Bizari D. (2019). Alginate-bioactive glass containing Zn and Mg composite scaffolds for bone tissue engineering. Int. J. Biol. Macromol..

[9] Neščáková Z., Zheng K., Liverani L., Nawaz Q., Galusková D., Kaňková H., Michálek M., Galusek D., Boccaccini A.R. (2019). Multifunctional zinc ion doped sol–gel derived mesoporous bioactive glass nanoparticles for biomedical applications. Bioact. Mater..

[10] Sánchez-Salcedo S., Shruti S., Salinas A.J., Malavasi G., Menabue L., Vallet-Regí M. (2014). In vitro antibacterial capacity and cytocompatibility of SiO 2-CaO-P2O5 meso-macroporous glass scaffolds enriched with ZnO. J. Mater. Chem. B.

[11] Paramita P., Ramachandran M., Narashiman S., Nagarajan S., Sukumar D.K., Chung T.W., Ambigapathi M. (2021). Sol–gel based synthesis and biological properties of zinc integrated nano bioglass ceramics for bone tissue regeneration. J. Mater. Sci. Mater. Med..

[12] Mouriño V., Cattalini J.P., Boccaccini A.R. (2012). Metallic ions as therapeutic agents in tissue engineering scaffolds: An overview of their biological applications and strategies for new developments. J. R. Soc. Interface.

[13] Yang H., Jia B., Zhang Z., Qu X., Li G., Lin W., Zhu D., Dai K., Zheng Y. (2020). Alloying design of biodegradable zinc as promising bone implants for load-bearing applications. Nat. Commun..

[14] Levy G.K., Goldman J., Aghion E. (2017). The prospects of zinc as a structural material for biodegradable implants—A review paper. Metals.

[15] Lin P.H., Sermersheim M., Li H., Lee P.H.U., Steinberg S.M., Ma J. (2018). Zinc in wound healing modulation. Nutrients.

[16] Pasquet J., Chevalier Y., Pelletier J., Couval E., Bouvier D., Bolzinger M.A. (2014). The contribution of zinc ions to the antimicrobial activity of zinc oxide. Colloids Surf. A Physicochem. Eng. Asp..

[17] Frígols B., Martí M., Salesa B., Hernández-Oliver C., Aarstad O., Ulset A.-S.T., Sætrom G.I., Aachmann F.L., Serrano-Aroca Á. (2019). Graphene oxide in zinc alginate films: Antibacterial activity, cytotoxicity, zinc release, water sorption/diffusion, wettability and opacity. PLoS ONE.

[18] Rembe J.D., Boehm J.K., Fromm-Dornieden C., Hauer N., Stuermer E.K. (2020). Comprehensive analysis of zinc derivatives pro-proliferative, anti-apoptotic and antimicrobial effect on human fibroblasts and keratinocytes in a simulated, nutrient-deficient environment in vitro. Int. J. Mol. Cell. Med..

[19] Tabata Y. (2009). Biomaterial technology for tissue engineering applications. J. R. Soc. Interface.

[20] Laurenti M., Cauda V. (2017). ZnO nanostructures for tissue engineering applications. Nanomaterials.

[21] Sabater i Serra R., Molina-mateo J., Torregrosa-cabanilles C., Andrio-Balado A., Meseguer Dueñas J., Serrano-Aroca A. (2020). Bio-Nanocomposite Hydrogel Based on Zinc Conformation, Thermal Behavior/Degradation, and Dielectric Properties. Polymers.

[22] Tiffany A.S., Gray D.L., Woods T.J., Subedi K., Harley B.A.C. (2019). The inclusion of zinc into mineralized collagen scaffolds for craniofacial bone repair applications. Acta Biomater..

[23] De Aragão Tavares E., De Medeiros W.M.T.Q., De Assis Pontes T.P., Barbosa M.M., De Araújo A.A., De Araújo R.F., Figueiredo J.G., Leitão R.C., Da Silva Martins C., Da Silva F.O.N. (2019). Chitosan membrane modified with a new zinc(II)-vanillin complex improves skin wound healing in diabetic rats. Front. Pharmacol..

[24] Priyadarshi R., Kumar B., Rhim J.W. (2020). Green and facile synthesis of carboxymethylcellulose/ZnO nanocomposite hydrogels crosslinked with Zn2+ ions. Int. J. Biol. Macromol..

[25] Ciofani G., Genchi G.G., Mattoli V. (2012). ZnO nanowire arrays as substrates for cell proliferation and differentiation. Mater. Sci. Eng. C.

[26] Lee J., Kang B.S., Hicks B., Chancellor T.F., Chu B.H., Wang H.T., Keselowsky B.G., Ren F., Lele T.P. (2008). The control of cell adhesion and viability by zinc oxide nanorods. Biomaterials.

[27] Jiménez M., Abradelo C., San Román J., Rojo L. (2019). Bibliographic review on the state of the art of strontium and zinc based regenerative therapies. Recent developments and clinical applications. J. Mater. Chem. B.

[28] Bae Y.S., Hill N.D., Bibi Y., Dreiher J., Cohen A.D. (2010). Innovative uses for zinc in dermatology. Dermatol. Clin..

[29] Azgın İ., Arbağ H., Eryılmaz M.A., Çelik Z.E. (2020). The effects of local and intraperitoneal zinc treatments on maxillofacial fracture healing in rabbits. J. Cranio-Maxillofac. Surg..

[30] Sharir H., Zinger A., Nevo A., Sekler I., Hershfinkel M. (2010). Zinc released from injured cells is acting via the Zn2+-sensing receptor, ZnR, to trigger signaling leading to epithelial repair. J. Biol. Chem..

[31] Wilson D., Varigos G., Ackland M.L. (2006). Apoptosis may underlie the pathology of zinc-deficient skin. Immunol. Cell Biol..

[32] Michaelsson G., Ljunghall K., Danielson B.G. (1980). Zinc in epidermis and dermis in healthy subjects. Acta Derm. Venereol..

[33] Pastar I., Stojadinovic O., Tomic-Canic M. (2008). Role of keratinocytes in healing of chronic wounds. Surg. Technol. Int..

[34] Lansdown A.B.G., Mirastschijski U., Stubbs N., Scanlon E., Ågren M.S. (2007). Zinc in wound healing: Theoretical, experimental, and clinical aspects. Wound Repair Regen..

[35] Holmes A.M., Mackenzie L., Roberts M.S. (2020). Disposition and measured toxicity of zinc oxide nanoparticles and zinc ions against keratinocytes in cell culture and viable human epidermis. Nanotoxicology.

[36] Li Y., Maret W. (2009). Transient fluctuations of intracellular zinc ions in cell proliferation. Exp. Cell Res..

[37] Yamamoto H., Tsuruoka S., Ioka T., Ando H., Ito C., Akimoto T., Fujimura A., Asano Y., Kusano E. (2006). Indoxyl sulfate stimulates proliferation of rat vascular smooth muscle cells. Kidney Int..

[38] Bozym R.A., Chimienti F., Giblin L.J., Gross G.W., Korichneva I., Li Y., Libert S., Maret W., Parviz M., Frederickson C.J. (2010). Free zinc ions outside a narrow concentration range are toxic to a variety of cells in vitro. Exp. Biol. Med. Maywood.

[39] Du Y., Guo D., Wu Q., Liu D., Bi H. (2014). Zinc chloride inhibits human lens epithelial cell migration and proliferation involved in TGF-β1 and TNF-α signaling pathways in HLE B-3 cells. Biol. Trace Elem. Res..

[40] Salazar-García S., García-Rodrigo J.F., Martínez-Castañón G.A., Ruiz-Rodríguez V.M., Portales-Pérez D.P., Gonzalez C. (2020). Silver nanoparticles (AgNPs) and zinc chloride (ZnCl2) exposure order determines the toxicity in C6 rat glioma cells. J. Nanopart. Res..

[41] Tekuri S.K., Bassaiahgari P., Gali Y., Amuru S.R., Pabbaraju N. (2021). Determination of Median Lethal Dose of Zinc chloride in Wistar Rat. Adv. Anim. Vet. Sci..

[42] Wang Y., Zhang Y., Guo Y., Lu J., Veeraraghavan V.P., Mohan S.K., Wang C., Yu X. (2019). Synthesis of Zinc oxide nanoparticles from Marsdenia tenacissima inhibits the cell proliferation and induces apoptosis in laryngeal cancer cells (Hep-2). J. Photochem. Photobiol. B Biol..

[43] Emri E., Miko E., Bai P., Boros G., Nagy G., Rózsa D., Juhász T., Hegeds C., Horkay I., Remenyik É. (2015). Effects of non-toxic zinc exposure on human epidermal keratinocytes. Metallomics.

[44] Lamore S.D., Cabello C.M., Wondrak G.T. (2010). The topical antimicrobial zinc pyrithione is a heat shock response inducer that causes DNA damage and PARP-dependent energy crisis in human skin cells. Cell Stress Chaperones.

[45] Lee S.H., Pie J.E., Kim Y.R., Lee H.R., Son S.W., Kim M.K. (2012). Effects of zinc oxide nanoparticles on gene expression profile in human keratinocytes. Mol. Cell. Toxicol..

[46] Sato R., Yamamoto H., Kasai K., Yamauchi M. (2002). Distribution pattern of versican, link protein and hyaluronic acid in the rat periodontal ligament during experimental tooth movement. J. Periodontal Res..

[47] Zimmermann D.R., Dours-Zimmermann M.T., Schubert M., Bruckner-Tuderman L. (1994). Versican is expressed in the proliferating zone in the epidermis and in association with the elastic network of the dermis. J. Cell Biol..

[48] Beikler T., Peters U., Prior K., Eisenacher M., Flemmig T.F. (2008). Gene expression in periodontal tissues following treatment. BMC Med. Genom..

[49] West J.J., Harris T.J.C. (2016). Cadherin Trafficking for Tissue Morphogenesis: Control and Consequences. Traffic.

[50] Hung C.F., Chiang H.S., Lo H.M., Jian J.S., Wu W. (2006). Bin E-cadherin and its downstream catenins are proteolytically cleaved in human HaCaT keratinocytes exposed to UVB. Exp. Dermatol..

[51] Yeh J.T., Yeh L.K., Jung S.M., Chang T.J., Wu H.H., Shiu T.F., Liu C.Y., Kao W.W.Y., Chu P.H. (2010). Impaired skin wound healing in lumican-null mice. Br. J. Dermatol..

[52] Niewiarowska J., Brézillon S., Sacewicz-Hofman I., Bednarek R., Maquart F.X., Malinowski M., Wiktorska M., Wegrowski Y., Cierniewski C.S. (2011). Lumican inhibits angiogenesis by interfering with α2β1 receptor activity and downregulating MMP-14 expression. Thromb. Res..

[53] Liu X.J., Kong F.Z., Wang Y.H., Zheng J.H., Wan W.D., Deng C.L., Mao G.Y., Li J., Yang X.M., Zhang Y.L. (2013). Lumican Accelerates Wound Healing by Enhancing α2β1 Integrin-Mediated Fibroblast Contractility. PLoS ONE.

[54] Handford P.A. (2000). Fibrillin-1, a calcium binding protein of extracellular matrix. Biochim. Biophys. Acta BBA Mol. Cell Res..

[55] Adamo C.S., Zuk A.V., Sengle G. (2021). The fibrillin microfibril/elastic fibre network: A critical extracellular supramolecular scaffold to balance skin homoeostasis. Exp. Dermatol..

[56] Philips N., Samuel M., Arena R., Chen Y.J., Conte J., Natrajan P., Haas G., Gonzalez S. (2010). Direct inhibition of elastase and matrixmetalloproteinases and stimulation of biosynthesis of fibrillar collagens, elastin, and fibrillins by xanthohumol. J. Cosmet. Sci..

[57] Dzamba B.J., Keene D.R., Isogai Z., Charbonneau N.L., Karaman-Jurukovska N., Simon M., Sakai L.Y. (2001). Assembly of epithelial cell fibrillins. J. Investig. Dermatol..

[58] Masaki H. (2010). Role of antioxidants in the skin: Anti-aging effects. J. Dermatol. Sci..

[59] Kruk J., Duchnik E. (2014). Oxidative stress and skin diseases: Possible role of physical activity. Asian Pac. J. Cancer Prev..

[60] Butt H., Mehmood A., Ali M., Tasneem S., Tarar M.N., Riazuddin S. (2019). Vitamin E preconditioning alleviates in vitro thermal stress in cultured human epidermal keratinocytes. Life Sci..

[61] Calcabrini C., De Bellis R., Mancini U., Cucchiarini L., Potenza L., De Sanctis R., Patrone V., Scesa C., Dachà M. (2010). Rhodiola rosea ability to enrich cellular antioxidant defences of cultured human keratinocytes. Arch. Dermatol. Res..

[62] Casares L., García V., Garrido-Rodríguez M., Millán E., Collado J.A., García-Martín A., Peñarando J., Calzado M.A., de la Vega L., Muñoz E. (2020). Cannabidiol induces antioxidant pathways in keratinocytes by targeting BACH1. Redox Biol..

[63] Salucci S., Burattini S., Buontempo F., Martelli A.M., Falcieri E., Battistelli M. (2017). Protective effect of different antioxidant agents in UVB-irradiated keratinocytes. Eur. J. Histochem..

[64] Son D.H., Yang D.J., Sun J.S., Kim S.K., Kang N., Kang J.Y., Choi Y.H., Lee J.H., Moh S.H., Shin D.M. (2018). A novel peptide, nicotinyl–isoleucine–valine–histidine (Na–IVH), promotes antioxidant gene expression and wound healing in HaCaT cells. Mar. Drugs.

[65] Yu L., Venkataraman S., Coleman M.C., Spitz D.R., Wertz P.W., Domann F.E. (2006). Glutathione peroxidase-1 inhibits UVA-induced AP-2α expression in human keratinocytes. Biochem. Biophys. Res. Commun..

[66] Rezvani H.R., Mazurier F., Cario-André M., Pain C., Ged C., Taïeb A., De Verneuil H. (2006). Protective effects of catalase overexpression on UVB-induced apoptosis in normal human keratinocytes. J. Biol. Chem..

[67] Miyachi Y. (1995). Photoaging from an oxidative standpoint. J. Dermatol. Sci..

[68] Dahle J., Kvam E., Stokke T. (2005). Bystander effects in UV-induced genomic instability: Antioxidants inhibit delayed mutagenesis induced by ultraviolet A and B radiation. J. Carcinog..

[69] Masaki H., Izutsu Y., Yahagi S., Okano Y. (2009). Reactive oxygen species in HaCaT keratinocytes after UVB irradiation are triggered by intracellular Ca^2+^ levels. J. Investig. Dermatol. Symp. Proc..

[70] Pluemsamran T., Onkoksoong T., Panich U. (2012). Caffeic acid and ferulic acid inhibit UVA-induced matrix metalloproteinase-1 through regulation of antioxidant defense system in keratinocyte HaCaT cells. In Proceedings of the Photochemistry and Photobiology. Photochem. Photobiol..

[71] Wu F., Cui L. (2017). Resveratrol suppresses melanoma by inhibiting NF-κB/miR-221 and inducing TFG expression. Arch. Dermatol. Res..

[72] Lee N.K. (2018). Preservation effects of geniposidic acid on human keratinocytes (HaCaT) against UVB. Biomed. Dermatol..

[73] Lim K.H., Ku J.-E., Rhie S.-J., Ryu J.Y., Bae S., Kim Y.-S. (2017). Anti-oxidant and Anti-inflammatory Effects of Sinapic Acid in UVB Irradiation-Damaged HaCaT Keratinocytes. Asian J. Beauty Cosmetol..

[74] Salesa B., Serrano-Aroca Á. (2021). Multi-Layer Graphene Oxide in Human Keratinocytes: Time-Dependent Cytotoxicity, Proliferation, and Gene Expression. Coatings.

